# BrainLiner: A Neuroinformatics Platform for Sharing Time-Aligned Brain-Behavior Data

**DOI:** 10.3389/fninf.2016.00003

**Published:** 2016-01-26

**Authors:** Makoto Takemiya, Kei Majima, Mitsuaki Tsukamoto, Yukiyasu Kamitani

**Affiliations:** ^1^Department of Neuroinformatics, ATR Computational Neuroscience LaboratoriesKyoto, Japan; ^2^Department of Information Science and Technology, Graduate School of Informatics, Kyoto UniversityKyoto, Japan

**Keywords:** data sharing, database, search, neuroscience, neuroinformatics, web service, machine learning, neural decoding

## Abstract

Data-driven neuroscience aims to find statistical relationships between brain activity and task behavior from large-scale datasets. To facilitate high-throughput data processing and modeling, we created BrainLiner as a web platform for sharing time-aligned, brain-behavior data. Using an HDF5-based data format, BrainLiner treats brain activity and data related to behavior with the same salience, aligning both behavioral and brain activity data on a common time axis. This facilitates learning the relationship between behavior and brain activity. Using a common data file format also simplifies data processing and analyses. Properties describing data are unambiguously defined using a schema, allowing machine-readable definition of data. The BrainLiner platform allows users to upload and download data, as well as to explore and search for data from the web platform. A WebGL-based data explorer can visualize highly detailed neurophysiological data from within the web browser, and a data-driven search feature allows users to search for similar time windows of data. This increases transparency, and allows for visual inspection of neural coding. BrainLiner thus provides an essential set of tools for data sharing and data-driven modeling.

## 1. Introduction

Data-driven science allows patterns in data collected from complex systems to be elicited without relying on explicit assumptions about the structure or interactions of elements within a system. In neuroscience, neural encoding and decoding based on data-driven prediction models have been shown to be useful approaches for revealing the neural representations of sensory, motor, and even subjective information (Pereira et al., 2009; Naselaris et al., 2011). Neural decoding approaches have demonstrated the efficacy of using statistical prediction models trained with brain activity associated with a task to decode subjective contents of task parameters (Kamitani and Tong, 2005), move robotic limbs (Wessberg et al., 2000; Hochberg et al., 2006, 2012; Schwartz et al., 2006), reconstruct visually presented stimuli (Miyawaki et al., 2008), and elicit the contents of dreams (Horikawa et al., 2013). Neural encoding approaches, on the other hand, have shown that brain activity can be matched to databases of images (Kay et al., 2008) and videos (Nishimoto et al., 2011).

While data-driven approaches have proven useful for revealing to some extent the structure of information representation in the brain, performing experiments is costly and time consuming, and often has an associated moral cost, such as when experiments result in the death or reduced lifespan of animals. To maximize the benefit of performing these experiments to society, it is imperative that researchers share their data openly to allow not only the validation of methodology, but also to enable other researchers to develop and test new data-driven algorithms that attempt to decipher the activity of the brain.

Often people who are experts in algorithms and data mining do not have backgrounds in neuroscience or access to facilities where they could perform experiments to collect data. To promote the development of new techniques of analyzing neurophysiological data, it is important to provide access to data sets across many conditions and recording modalities. This can lead to the development of new algorithms that can find patterns and trends in data without relying on large amounts of domain knowledge. This may help solve many problems in neuroscience, including uncovering the neural correlates of consciousness (Poline et al., 2012).

Many platforms for neurophysiological data sharing exist, such as CRCNS^1^ (Teeters and Sommer, 2009), the INCF G-Node^2^ (Herz et al., 2008), INCF Data Space^3^, INCF Japan Node^4^, Neurotycho^5^, EEGBase ^6^, OpenfMRI^7^, CARMEN^8^, and NeuroVault^9^ (Gorgolewski et al., 2015), but none of these focuses on explicitly providing behaviorally aligned brain activity data for assisting data driven neuroscience and neural decoding. To remedy this, we implemented http://BrainLiner.jp, as a platform to facilitate data sharing for neuroscience. The BrainLiner platform not only provides a way to freely share data among researchers, but also allows researchers to perform a data-driven similarity search.

In this contribution, we present the BrainLiner platform for sharing neurophysiological data, our schema-based data format, data explorer, and our unsupervised, data-driven similarity search algorithm. Our search algorithm extracts spectral features from data at varying temporal resolutions and calculates the Pearson correlation to quantify the similarity between two time points within files shared on BrainLiner. Verifying the search algorithm on rat electrocorticographic data demonstrates that the algorithm can find brain activity that corresponds to a similar behavioral task.

## 2. The Brainliner platform

**Figure 1:**
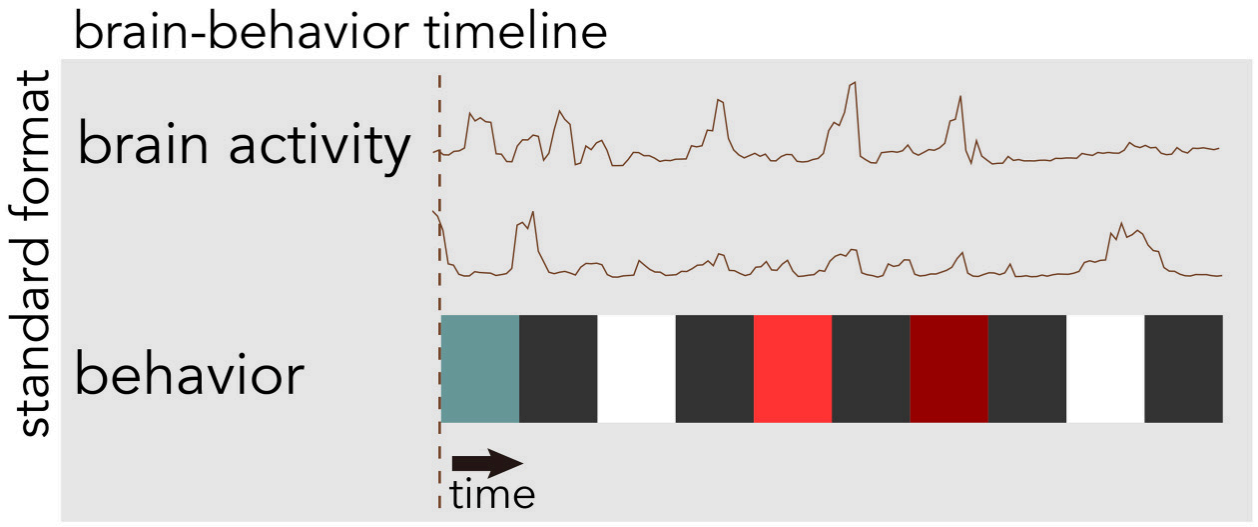
**The BrainLiner platform combines brain activity and behavioral data that are time-aligned on the same axis**. In this example, behavioral data are represented as colored blocks, where each block is a different task condition; data in the figure are schematic.

BrainLiner (http://BrainLiner.jp) is an online web portal for sharing, as well as searching time-aligned neurophysiological and stimulus/behavioral data. The name *BrainLiner* emphasizes the focus on supporting data-driven neuroscience by sharing brain activity data that are time-aligned with data about the task and behavior of the subject from which the data were recorded, as in Figure 1. For example, if a human subject viewed images presented at 3-s intervals with 64-channel electrocorticography (ECoG) recording, then information about the images presented at each time point should be aligned with the ECoG brain activity data; data about the task and behavior are of equal importance.

**Figure 2:**
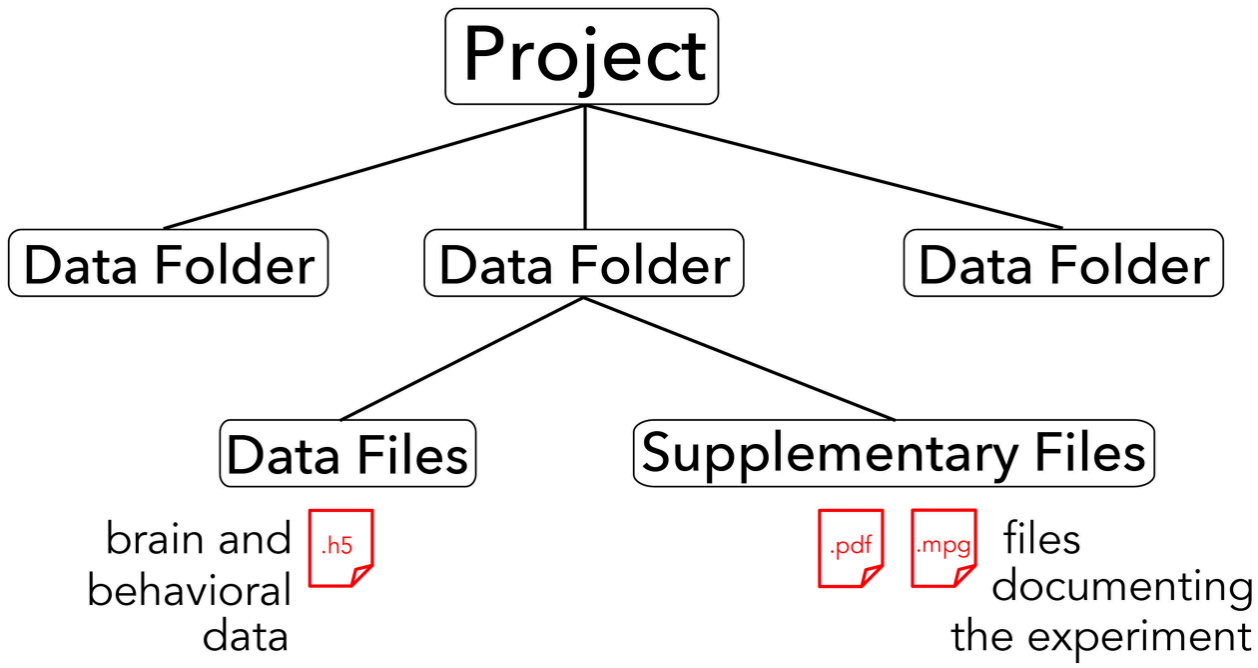
**Hierarchical structure of BrainLiner projects**. Files and information about experimental hardware and subjects are grouped into data folders. Data files must be in .h5 format, whereas supplementary files can be any arbitrary data form. Data folders are grouped into projects.

The core of the BrainLiner platform is the portal at http://BrainLiner.jp. This was programmed from scratch using Java for the server, mySQL to store metadata, and HDF5 to store project data. Researchers can log into the web portal via their Google account, without needing to register another username and password to remember. Once logged in, users can upload data and documents describing their data and experimental conditions. All data are organized into projects and folders. As shown in Figure 2, data files are uploaded to folders within projects and published papers related to the data can be associated with a project. Data files are arranged in a common format and are further decomposed into groups of brain activity and behavioral data (Section 3).

**Figure 3:**
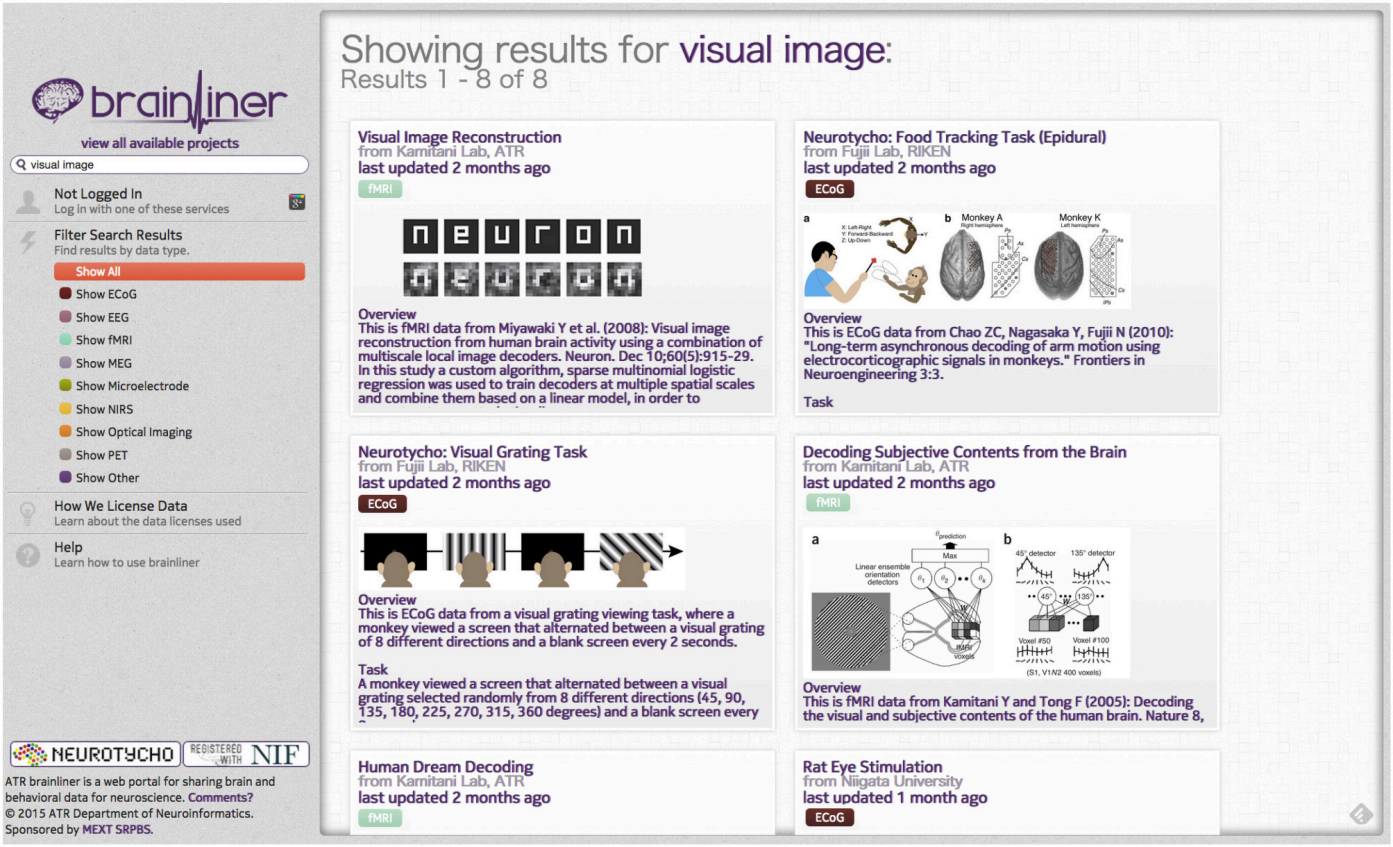
**Result screen of the BrainLiner platform for a text-based search query**.

On the BrainLiner web portal, users can execute text-based search queries against indexed text data, as shown in Figure 3. Text data that are searchable on BrainLiner include project titles and descriptions, descriptions inside data files, and text within supplementary files, such as Portable Document Format (PDF) files. Text-based information retrieval is a relatively well-understood field, and indeed many open source libraries exist for text search (BrainLiner uses Hibernate Search^10^ with Apache Lucene^11^). In addition to textual search, BrainLiner also supports data-driven similarity search, described in Section 4, where users can search for similar data within data files.

If users find a project that interests them, they can preview data files in the project in order to gain an understanding of the type of data available. Previewing data can be done within the web browser, using the WebGL data explorer, shown in **Figure 7A**. The data explorer can be used to grasp whether a data file is of interest to a user without the need to download a file and view it locally. Even without registering an account or logging in, anyone can search for, explore, and download data.

## 3. Brainliner data format

All data files on BrainLiner use a common data format. This enables efficient data analysis because once the format of one file is understood, all data files available on BrainLiner can then be easily used without having to learn a new structure for each file. The data visualization and search features take as input files in the BrainLiner data format, so uploaded data files must be in the correct format when uploading.

One of the main objectives of the BrainLiner platform is to enable neural decoding approaches. To achieve reliable decoding results, contemporary machine learning requires large amounts of labeled data. The common data format used for BrainLiner was created in accordance with the philosophy that brain activity and behavioral data should be given equal salience and should also be aligned on a common time axis. The BrainLiner format is based on a schema of defined terms^12^, and as such, users can annotate their data with meta information that unambiguously describes the structure and type of data, such as the task (e.g., stimulus task) or modality of recording data [e.g., electrocorticography (ECoG) data]. This standardized, schema-based data format is based on Hierarchical Data Format 5 (HDF5)^13^, allowing files downloaded from BrainLiner.jp to be readable in a variety of programming languages and environments, such as MatLab, Python, R, C++, and Java, and across many different platforms. HDF5 also supports data compression, allowing files to be transmitted quickly over the Internet.

**Figure 4:**
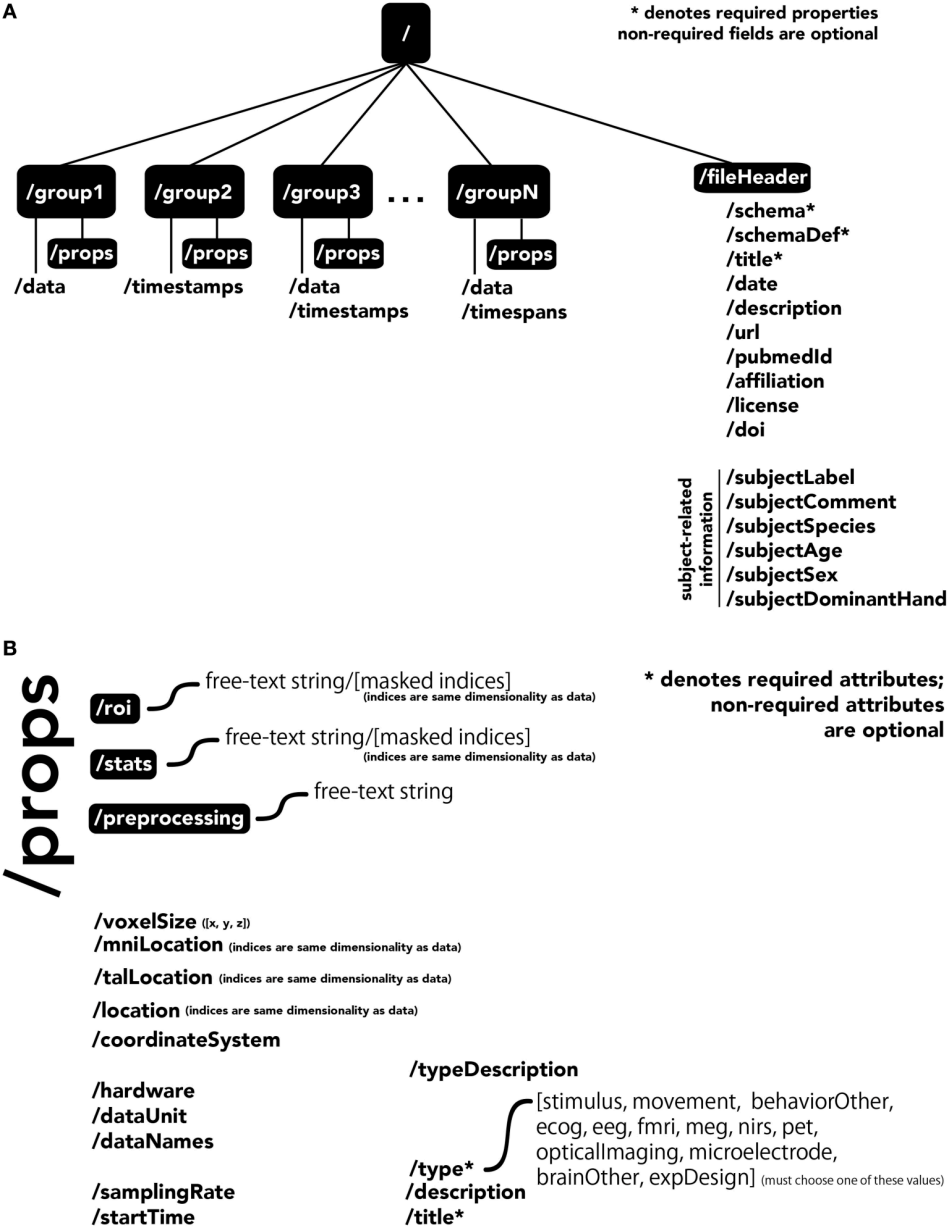
**The BrainLiner common data format**. Filled, black boxes denote HDF5 groups, words proceeded by a “/” denote HDF5 properties. **(A)** A file can contain multiple “groups,” each of which has an HDF5 dataset called “data,” “timespans,” and/or “timestamps.” **(B)** Users can specify properties for each “group” in “props.”

The central unit of organization within BrainLiner data files is called a group. As shown in Figure 4A, a data file can have many groups, each attached to the root of the file. Each group can have an HDF5 dataset called *data, timespans*, and/or *timestamps*. Whereas, the former can be used to store time-series data, such as brain activity or behavioral movement data, the *timespans* group can be used to store an array of starting and ending times, and the *timestamps* group can be used to store an array of timestamps, such as the timings of neural spikes or instantaneous events.

Users can specify properties associated with a group, by creating a sub-group called *props*. Properties available in the BrainLiner schema are shown in Figure 4B, and follow the camel-case naming convention (e.g., samplingRate). Users are free to choose from the pre-defined properties and only include the properties they need to describe their data. The only properties that are required are the *title* and *type* properties. The *type* property defines the modality of the brain activity recording equipment (e.g., fMRI, ECoG, etc.) or task (e.g., stimulus or physical movement). Information about the type of data in a dataset can enable automated processing of the data by software to do advanced functions, such as meta-analyses, adapting the display of data, or data-driven search. Each property defined in *props* is an HDF5 dataset, where the valid names of properties are defined in the BrainLiner schema or a custom schema provided by the user. Under the *props* group, *roi* and *stats* groups can also be optionally created, and contain HDF5 datasets with any arbitrary name, with the same dimensions as the *data* dataset. To support data provenance, free text describing how the data were created and processed can be explained under the *preprocessing* group.

In addition, the file itself can have properties associated with the root, located under the *fileHeader* group. These properties describe the experiment and also the subject the data was collected from.

**Figure 5:**
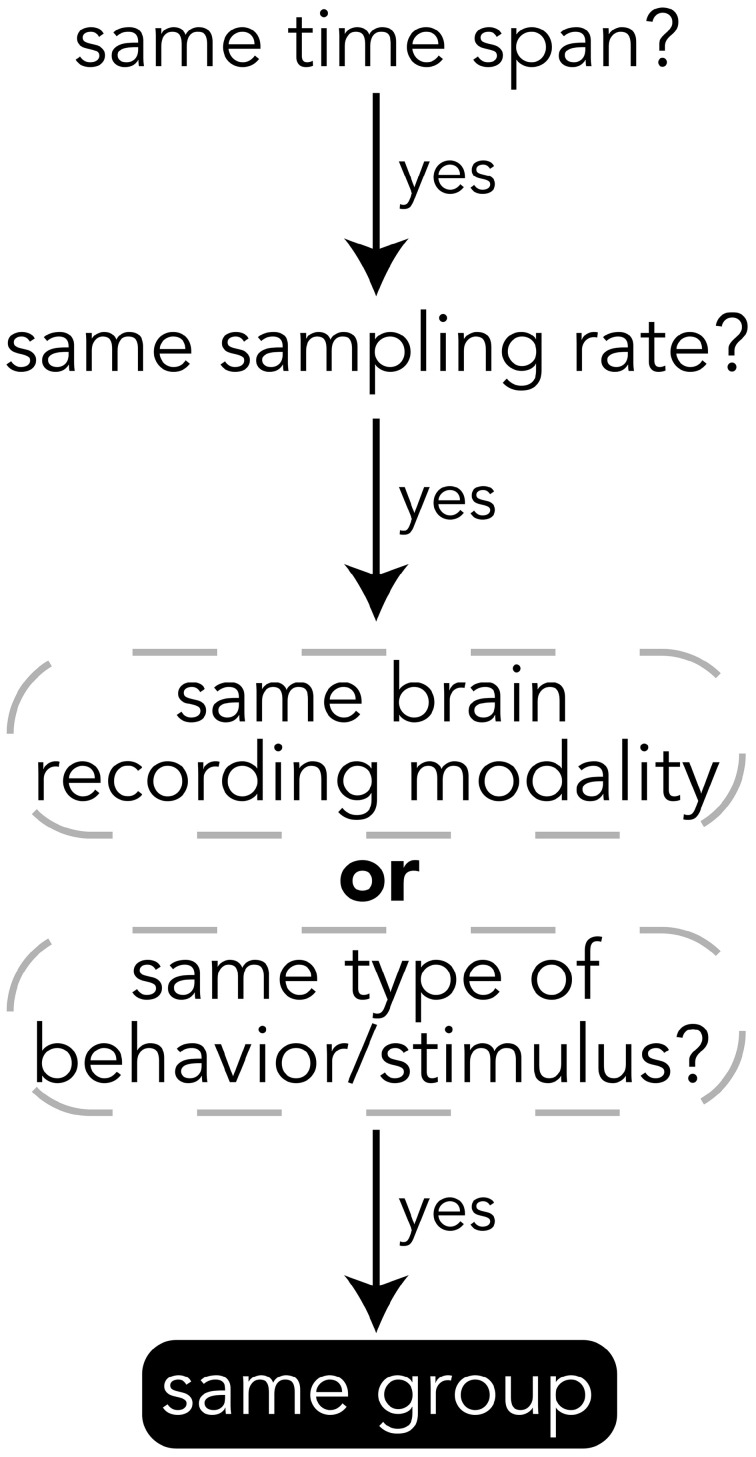
**Heuristics for grouping data into groups within a file**. While the grouping of data is up to the file creator, it is recommended to follow these heuristics.

When deciding how to group experimental data into groups, it is important to choose data that have the same temporal and semantic structure. Figure 5 shows a flow chart for deciding whether data should be in the same group or if a new group should be created for a set of data. The specifics of how data are divided into groups is up to the creator of the file, but a good heuristic for grouping data is that if data have the same time span, sampling rate, and are of either the same brain recording modality (if brain data) or are data for the same type of behavior or stimulus (e.g., visually presented stimuli), then the data should be in the same group. The data explorer (Section 4.1.4) and data search feature (Section 4) depend on the structure of the files to automatically create preview files and search indices, so the design of how data are structured is important for having meaningful results.

All data on BrainLiner are in a common file format, facilitating data processing and meta-analyses. Files are all in the standard HDF5 format, meaning that once data processing scripts or programs have been written once, they should be extensible to all the files on BrainLiner with a similar structure. To create files in the BrainLiner format, users should first decide how to group their data according the heuristics in Figure 5 and then construct an HDF5 file according to the format in Figure 4. Creating HDF5 files can be done in a variety of programming languages using libraries such as *H5py*^14^ in Python, for example. Example code for creating and reading BrainLiner format HDF5 files an be found at https://github.com/ATR-DNI/BrainLinerDataFormat and we encourage users to upload and share their file processing and analysis code with the community.

### 3.1. Data licensing

When users upload data to BrainLiner, they can license data in one of three ways: (1) Open Database Commons Public Domain and Dedication License (PDDL)^15^, (2) Open Database Commons Attribution License (ODC-BY) ^16^, or (3) choose a custom license, where users can write any arbitrary license for their data. Licenses are described as follows.

- **Open Database Commons Public Domain and Dedication License (PDDL)**. PDDL is similar to the well-known CC-0 license (https://creativecommons.org/publicdomain/zero/1.0/), in that users licensing their data under PDDL put the data out freely without any restrictions whatsoever. This allows creative uses of data that the original author may not have envisioned.
- **Open Database Commons Attribution License (ODC-BY.)** ODC-BY allows anyone to freely use uploaded data, with the only restriction being that data have to be cited when used. Users who choose this license can specify exactly how they want to be cited. This is useful for cases when uploaded data are related to a published study and it is desirable that anyone who uses the data cites the study.
- **Custom License**. For special cases where PDDL or ODC-BY will not work, users can write their own license and apply it to their data.

## 4. Data similarity search

To allow people to find patterns in data files, we created an unsupervised data similarity search feature. The targeted use case for this feature is for a user to both confirm that a data file is of high quality and to explore a data file to find interesting vectors for analysis. The design and implementation details are explained in subsequent sections and Section 4.2 provides some validation for our method.

As described in the previous section, having a consistent, common data format can enable advanced, automated analyses of data files on BrainLiner. This includes the ability to extract features, index, and search data, enabling data-driven search. Recently, the ability to retrieve data similar to given input data has been brought to the mainstream through image search (Shrivastava et al., 2011) and music search (Wang, 2003). Whereas, text-driven search often exploits knowledge of natural language syntax and grammar (Jackson and Moulinier, 2007), data-driven search often relies on the structure of the data being targeted for information retrieval. For example, many image-based search methods extract features from images and use those to form an input query to find similar features (Batko et al., 2010). Music search tools exploit patterns in spectral powers of songs (Wang, 2003) in order to quickly find similar data.

Because BrainLiner aims to support neural decoding, we want to enable data-driven similarity search in order to quantify the similarity of neural activity related to behavioral data within shared files. Previous work by Tomen et al. (2012) quantized features extracted from spectral powers into binary vectors that could then be quickly compared via dice coefficients. While this approach could search data quickly, quantizing the data into binary vectors was a memory- and computationally expensive process. Therefore, we reconsidered how to implement the data-driven similarity search, focusing on developing a method that can find task-related data in an unsupervised way, while achieving good performance with respect to memory and computational complexity.

While contemporary deep learning approaches can use techniques such as convolutional neural networks to exploit spatially correlated patterns to retrieve similar images without explicitly specifying the features used (Krizhevsky et al., 2012) or to do visual object recognition (Cadieu et al., 2014), these approaches require considerably large amounts of data and computational resources. Therefore, the BrainLiner data search feature was designed and implemented to quickly do a similarity comparison between time windows, without needing to process large amounts of data to enable searching.

### 4.1. Design and implementation

**Figure 6:**
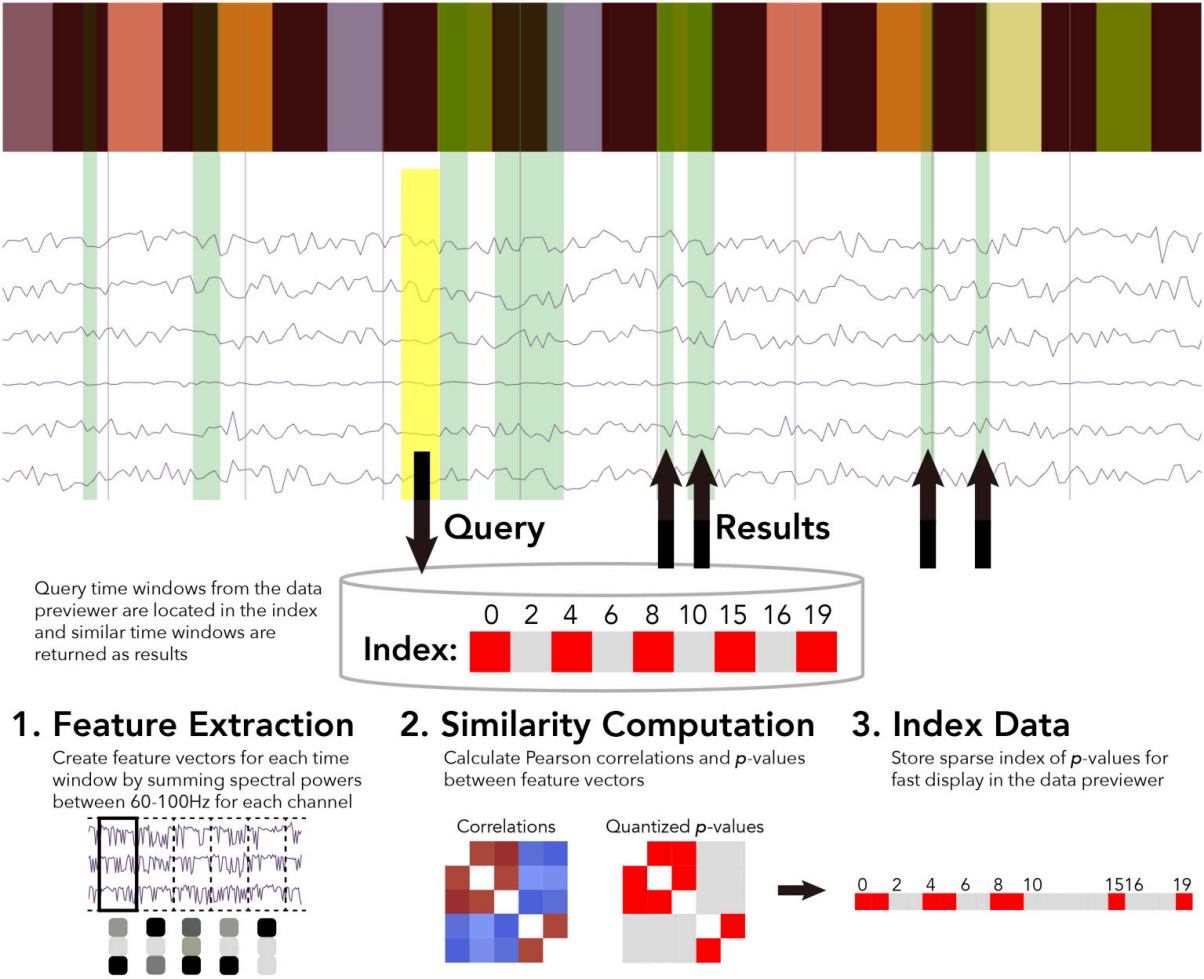
**WebGL data explorer showing task stimulus and the first six channels of a data file (top) and the preprocessing steps for the data-driven similarity search algorithm (bottom)**. The input search query is shown as a yellow rectangle and the returned results are displayed as green rectangles in the data explorer. The bar of colored rectangles at the top of the data explorer denotes a behavioral data channel, with each colored rectangle representing a stimulus of a different type, with the same types being the same color.

For the BrainLiner data similarity search, we decided to focus primarily on electrocorticography (ECoG) data [though electroencephalography (EEG) data are also supported]. With performance in mind, we implemented an unsupervised, data-driven similarity search tool in the BrainLiner data explorer that can find similar time windows of data for given, input time windows. Figure 6 shows an overview of the BrainLiner data-driven similarity search algorithm. Via the web interface, first a user views a file using the data explorer (described in Section 2). Next, the user selects a time span of data in the previewer as the input query, consisting of a start and an end time. The query is then looked up in the index of data for the file, and all time windows of data within the file that meet our similarity criteria (see Section 4.1.2) are returned and displayed in the data explorer.

#### 4.1.1. Feature extraction

First, for each file, ECoG and EEG data channels are split up into time windows spanning the entire duration of the recording. Four time window durations were considered: 500 ms, 1, 1.5, and 5 s. Time windows for each time scale were grouped into separate sets. For each time window, spectral powers were calculated using the Fast Fourier Transform (FFT). High gamma spectral powers between 60 and 100 Hz were then summed for each time window and z-score normalized for each ECoG and EEG electrode channel.

Thus, for each time window of data in a file, the number of features extracted is exactly *n*, where *n* is the number of channels. This has the effect of greatly compressing the data used in the similarity calculation. For example, 1 s of 64-channel ECoG data recorded at 10,000 Hz would be only a single length-64 vector of numbers for the 1 s time resolution, instead of 64,000 numbers.

Once the features are extracted from data for a file for all time windows, the pair-wise similarity between all time windows is then calculated.

#### 4.1.2. Similarity computation

After feature extraction, the data within each time window are represented as an *n*-length feature vector, where *n* is the number of ECoG and/or EEG channels within a file. For all pairs of time windows at each of the five time resolutions, the similarity between the feature vectors is calculated as the Pearson correlation between the two vectors. While calculating correlations between all time windows is a computationally intensive task, this only ever has to be done once for each file, as the results are stored in an index.

#### 4.1.3. Data indexing

After calculating the pairwise similarity between all time windows of ECoG and EEG data, the similarities between time windows are indexed for each file. Due to the large amount of data on BrainLiner, correlations between every time window for each file cannot be feasibly stored in memory for quick retrieval. To get around this, *p*-values are calculated for the Pearson correlations and data are stored sparsely as spans of *p*-values. As shown in Figure 6, the index consists of two lists: *p*-values and starting indices for time windows. To further reduce the data being searched, only positive correlations are stored in the index.

These enhancements allow the size of the index to grow linearly at *O*(*n*) in the worst case where every other time window is correlated with a two-tailed *p* ≤ 0.05. In practice the index size increases much lower than the linear case, as related brain activity tends to occur in temporal clusters. By using pre-defined sizes for time windows for which correlations are calculated, it is possible that there could be a bias to high correlations for some pairs of time windows based on the temporal structure of the task. However, by binary quantizing the results as significantly correlated or not based on the estimated *p*-value, small biases should not change the outcome of the results being displayed.

Because we calculate the Pearson correlation between all pairs of time windows, in the future we hope to expose the raw correlation values via a programmatic API so that programs can take advantage of the similarity search to enable complex analyses. Future work should also consider using information about task structure in the indexing and search process in a semi-supervised manner.

#### 4.1.4. User interface and data explorer

The data search was implemented as an asynchronous REST web service in Python using Tornado^17^. The REST interface takes as input the unique file identifier, the start time, and length of the query. From the length, the time scale for which results are returned is automatically determined. That is, the time scale that is the largest, yet still less than or equal to the length of the query, is used. For example, if the query length is 600ms, then the 500ms time scale is used.

**Figure 7:**
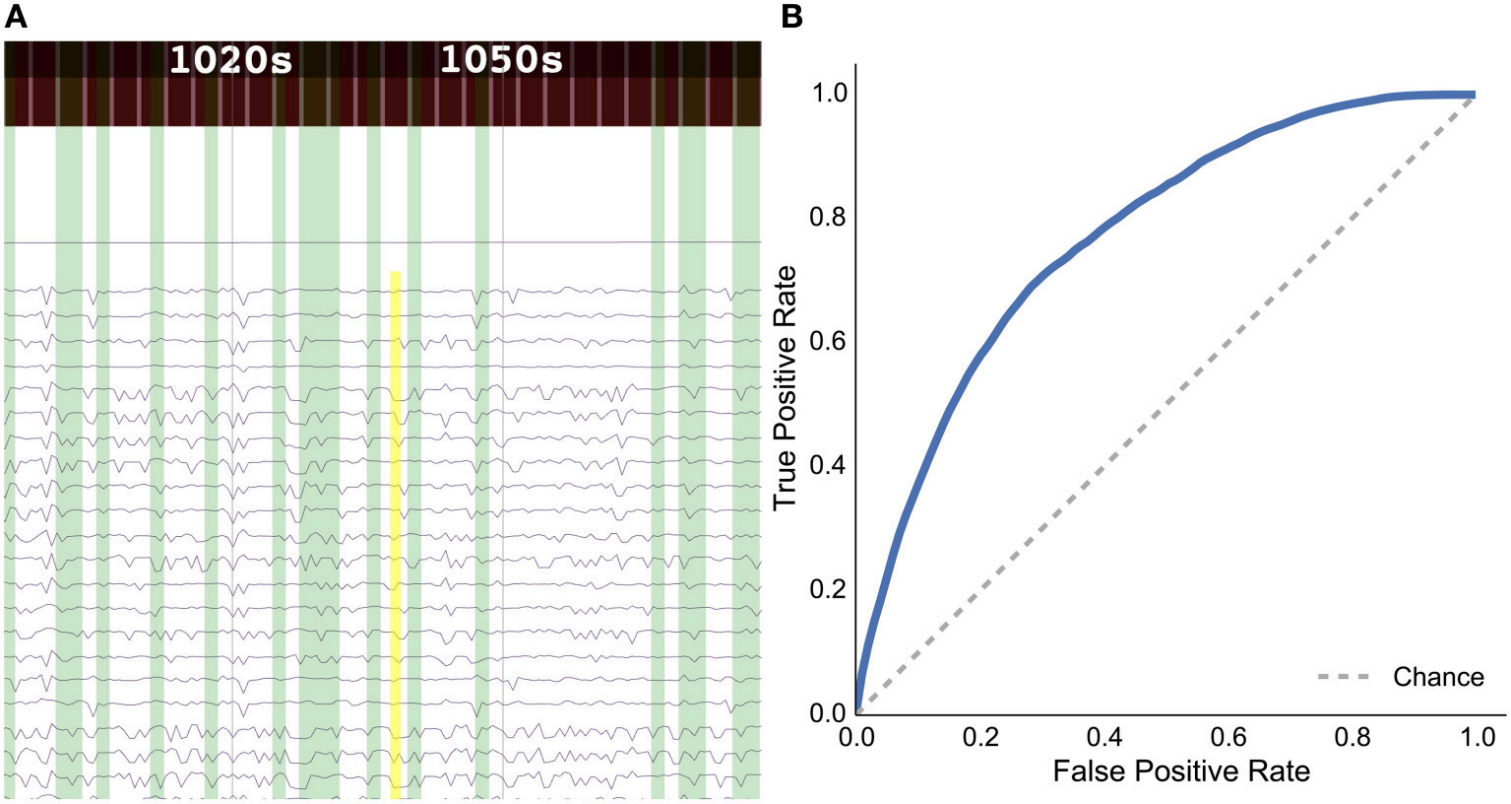
**Results for the rat experiment: (A) data explorer showing a search query (yellow rectangle) and results (green rectangles) for the session 1 file for rat LE010, and (B) ROC curve for rat LE010 (dashed line denotes chance level)**.

The user interface of the data explorer was implemented using vispy.js^18^, which uses WebGL to display hundreds of thousands of data points in real time. Users can zoom in and out with their mouse, and also freely pan the data. Figure 7A shows an example of the data explorer. When in search mode, the user can click and draw an input query (yellow) and the results will be shown in green. Because time series data are the input to the search, the search query is only drawn on top of time series data groups, whereas the results are drawn from the top to the bottom of the screen, in order to allow people to find and explore connections between task and behavioral information. The bars at the top of the figure shows different stimulus conditions and recorded behavior. These are time-aligned with the time series brain activity data, so in the data explorer, the user can view both the behavioral and task data along with the brain activity data.

If a file is searchable (has a data search index), then automatically a large “search” button and a “search help” button will be displayed. Clicking on the search button enables the data search mode. From there, a user can click and drag to select time windows of data, which will automatically be queried for search and the results will be displayed as green time windows that overlay the time series data in a file.

### 4.2. Data search validation and results

As a sample demonstration of the efficacy of the data search method for finding similar brain activity related to a task, we used data from Toda et al. (2011)^19^, where a rat had its eyes stimulated with a visual grating, while a 32-channel ECoG array recorded brain activity. For session 1 of rat LE010, there were 1148 time windows of 1.5 s. We calculated the pairwise correlation (Pearson *r*) between all pairs of time windows of 1.5 s. Each time window was then, in turn, set as an input query time window, and we then used scikit learn (Pedregosa et al., 2011)^20^ to calculate the true positive and false positive rates for the query, by varying the threshold for the correlations with all the other time windows. The true positive rate was defined as $\frac{TP}{\left(TP+FN\right)}$, where *TP* is the number of true positives and *FN* is the number of false positives; the false positive rate was $\frac{FP}{\left(FP+TN\right)}$, where *FP* is the number of false positives and *TN* denotes the number of true negatives. All the results for each query time window were then averaged. This was done to simulate what real queries would return, for each time window of data. The receiver-operating characteristic (ROC) curve for the averaged false positive and true positive rates is shown in Figure 7B.

The results show that the unsupervised, data-driven similarity search algorithm can retrieve time windows of the same task as an input query time window, suggesting that the search algorithm uses features that are relevant to experimental task. While this example is very simple and further analyses should be done with more data sets in the future, it does show the potential for the data driven search as being able to retrieve similar time windows of data.

## 5. Discussion

To help drive the progress of data-driven neuroscience approaches, the BrainLiner platform was created to share time-aligned brain and behavioral data on a unified time line. BrainLiner gives equal salience to both brain activity and data about the behavior and/or task, which can help researchers train statistical models to learn how brain activity represents stimuli and gives rise to behavior. Toward this end, data from previous neural decoding experiments are publicly available on BrainLiner, all in a standardized file format that allows one to readily process the files.

The new, schema-based data format used on BrainLiner offers both flexibility to people creating data files and well-defined meaning. This can facilitate automatic data analyses because once programs are written to process the format, all the files on the web portal can be processed in the same way. In the future, NIF ontologies^21^ could potentially be linked to the schema definitions, which will allow knowledge-based software to do automatic inference and find new connections inside data.

The WebGL data explorer allows people to find interesting patterns in data from within a web browser. An unsupervised, data-driven similarity search allows users to find similar time windows of data within a file. Brain activity, behavioral, and task data are all shown in a unified view within the data explorer, uniting all the traditionally separated data together.

The data-driven similarity search that is incorporated into the WebGL data explorer is a new feature created for BrainLiner. It allows people to discover patterns within a single data file, which may lead to ideas for new types of analyses.

Right now high-gamma spectral powers alone are used for the search, which work well for ECoG data, but may have trouble with EEG data because of noise within the frequency bands used. Future work should study pertinent features from a large database and try to incorporate different features for EEG and ECoG. Future work should also expand the search method across multiple files. Cross-file search should be readily implementable for fMRI, as fMRI data can be converted to standard brain spaces like MNI and then activity can be compared at the same coordinates in the brain. Comparing features across data of other modalities will require further research.

## Author contributions

YK conceived and directed the project. M. Takemiya designed the data format, created the web portal, and conducted data analysis. M. Tsukamoto and KM performed data conversion and analysis.

## Funding

Strategic Research Program for Brain Science (MEXT), the Ministry of Internal Affairs and Communications (“Novel and innovative R&D making use of brain structures”), ImPACT Program of Council for Science, Technology and Innovation (Cabinet Office, Government of Japan), MEXT KAKENHI Grant Number 26119536, 15H05920, JSPS KAKENHI Grant Number 26242088, 15H05710, and JST/DFG/BMBF collaborative research project in Computational Neuroscience.

### Conflict of interest statement

The authors declare that the research was conducted in the absence of any commercial or financial relationships that could be construed as a potential conflict of interest.

## Footnotes

^1^http://crcns.org/

^2^http://www.g-node.org/

^3^http://www.incf.org/resources/data-space

^4^http://www.neuroinf.jp/

^5^http://neurotycho.org/

^6^https://eegdatabase.kiv.zcu.cz/home-page

^7^https://openfmri.org/

^8^http://www.carmen.org.uk/

^9^http://neurovault.org/

^10^http://hibernate.org/search/

^11^http://lucene.apache.org/

^12^http://brainliner.jp/1.0

^13^http://www.hdfgroup.org/

^14^https://github.com/h5py/h5py

^15^http://opendatacommons.org/licenses/pddl/1.0/

^16^http://opendatacommons.org/licenses/by/

^17^http://www.tornadoweb.org/

^18^https://github.com/vispy/vispy.js

^19^http://brainliner.jp/data/brainliner/Rat_Eye_Stimulation

^20^http://scikit-learn.org/stable/modules/generated/sklearn.metrics.roc_curve.html

^21^http://www.neuinfo.org/
